# Proteomic Analysis Identifies Molecular Players and Biological Processes Specific to SARS-CoV-2 Exposure in Endothelial Cells

**DOI:** 10.3390/ijms231810452

**Published:** 2022-09-09

**Authors:** Thatiana Corrêa de Melo, Dilza Trevisan-Silva, Miryam P. Alvarez-Flores, Renata Nascimento Gomes, Marcelo Medina de Souza, Hellen Paula Valerio, Douglas S. Oliveira, Carlos DeOcesano-Pereira, Viviane Fongaro Botosso, Soraia Attie Calil Jorge, Mirta Schattner, Ricardo M. Gomez, Ana Marisa Chudzinski-Tavassi

**Affiliations:** 1 Centre of Excellence in New Target Discovery (CENTD), Butantan Institute, São Paulo 05503900, Brazil; 2 Virology Laboratory, Butantan Institute, São Paulo 05503900, Brazil; 3 Viral Biotechnology Laboratory, Butantan Institute, São Paulo 05503900, Brazil; 4 Laboratory of Experimental Thrombosis, Institute of Experimental Medicine (IMEX-CONICET-ANM), Buenos Aires 1425, Argentina; 5 Laboratory of Animal Viruses, Institute of Biotechnology and Molecular Biology, CONICET-UNLP, La Plata 1900, Argentina

**Keywords:** SARS-CoV-2, endothelial cells, proteomics, mass spectrometry, HUVECs

## Abstract

Severe acute respiratory syndrome coronavirus 2 (SARS-CoV-2) has been responsible for the severe pandemic of acute respiratory disease, coronavirus disease 2019 (COVID-19), experienced in the 21st century. The clinical manifestations range from mild symptoms to abnormal blood coagulation and severe respiratory failure. In severe cases, COVID-19 manifests as a thromboinflammatory disease. Damage to the vascular compartment caused by SARS-CoV-2 has been linked to thrombosis, triggered by an enhanced immune response. The molecular mechanisms underlying endothelial activation have not been fully elucidated. We aimed to identify the proteins correlated to the molecular response of human umbilical vein endothelial cells (HUVECs) after exposure to SARS-CoV-2, which might help to unravel the molecular mechanisms of endothelium activation in COVID-19. In this direction, we exposed HUVECs to SARS-CoV-2 and analyzed the expression of specific cellular receptors, and changes in the proteome of HUVECs at different time points. We identified that HUVECs exhibit non-productive infection without cytopathic effects, in addition to the lack of expression of specific cell receptors known to be essential for SARS-CoV-2 entry into cells. We highlighted the enrichment of the protein SUMOylation pathway and the increase in SUMO2, which was confirmed by orthogonal assays. In conclusion, proteomic analysis revealed that the exposure to SARS-CoV-2 induced oxidative stress and changes in protein abundance and pathways enrichment that resembled endothelial dysfunction.

## 1. Introduction

The 2019 global health emergency of coronavirus disease 2019 (COVID-19), caused by the novel human coronavirus, severe acute respiratory syndrome coronavirus 2 (SARS-CoV-2), has resulted in more than six million deaths worldwide (https://covid19.who.int; accessed on 7 July 2022) [1]. Global efforts are still ongoing to develop effective vaccines and new therapeutic agents to mitigate the pandemic and prevent the re-emergence of COVID-19. In the continuous cycles of transmission, different SARS-CoV-2 variants have emerged posing a threat to overturn the significant progress made so far in limiting the spread of this virus [2,3]. In severe COVID-19 cases, the most common clinical manifestation is acute pneumonia with ground-glass opacities on computed tomography [4,5]. More recently, the severity and mortality of COVID-19 have been shown to positively correlate with coagulopathy, thrombosis, and D-dimer levels, contributing to a hyperimmune response [6]. The pathogenesis of COVID-19 is associated with vasculopathy with micro- and macrothrombotic lesions in the lungs and other organs [7]. Vascular endothelial dysfunction involves inflammation, which leads to endothelitis [6,8]. Although these findings provide the basis for better classification of COVID-19 as a thromboinflammatory disease [8], the cellular mechanisms by which SARS-CoV-2 induces endothelial damage remain to be investigated. The vascular endothelium is composed of endothelial cells (ECs) that form a barrier between the vessels and tissues. This barrier is responsible for regulating distinct processes, including the modulation of vascular tone, dynamic permeability, thrombogenicity, inflammation, angiogenesis, and maintenance of vascular homeostasis [9,10]. The entry of SARS-CoV-2 into host cells depends on a sequence of steps involving the interaction of the viral spike (S) protein with host-cell membrane receptors [11]. Entry mediated by the S protein, angiotensin-converting enzyme 2 (ACE2) receptor, and proteases, such as transmembrane serine protease 2 (TMPRSS2), are well-characterized [12,13]. ACE2 is differentially expressed in different human tissues and cells, and its weak interaction with the S protein and ACE2 receptor suggests distinct susceptibility to SARS-CoV-2 infection [11]. Recent studies showed the importance of integrins for SARS-CoV-2 entry in ACE-2 negative endothelial cells [14,15]. It is suggested that SARS-CoV-2 interacts with αvβ3 integrins via the RGD motif of the S protein and the entry into cells is mediated by endocytosis [14]. It is still unclear whether there are other pathways by which the virus enters target cells [16,17]. Some advances were made in the identification of host-cell proteins that modulate virus replication in infected cells. Knockout of SAMHD1 in 293T and differentiated THP1 cells increased innate immune response and suppressed SARS-CoV-2 replication with a prominent increase in *STAT1* mRNA levels [18]. Genome-wide CRISP-Cas9 screen and integrative analysis highlighted new pro- or anti-viral genes, four of them were validated and negatively affect the SARS-CoV-2 replication: *ATP6V0D1*, *DAZAP2*, *VTA1*, and *KLF5* [19].

There is evidence in the literature that coagulation dysfunction and vascular pathology in COVID-19 are due to the direct infection of endothelial cells [20,21,22]. On the contrary, some studies have indicated that COVID-19 coagulopathy is a response to circulating mediators produced in the microenvironment cells infected with SARS-CoV-2 and is not directly related to the infection of the endothelium by SARS-CoV-2 [6,10,23]. In order to address these inconsistent findings and to contribute to our understanding of the cellular mechanisms involved in endothelial injury, we investigated the global proteomic response of human umbilical vein endothelial cells (HUVECs) after exposure to SARS-CoV-2. We were able to identify changes in the abundance of specific proteins and the enrichment of cellular pathways that clearly show responses to cell stress and endothelial damage.

## 2. Results

**Figure 1:**
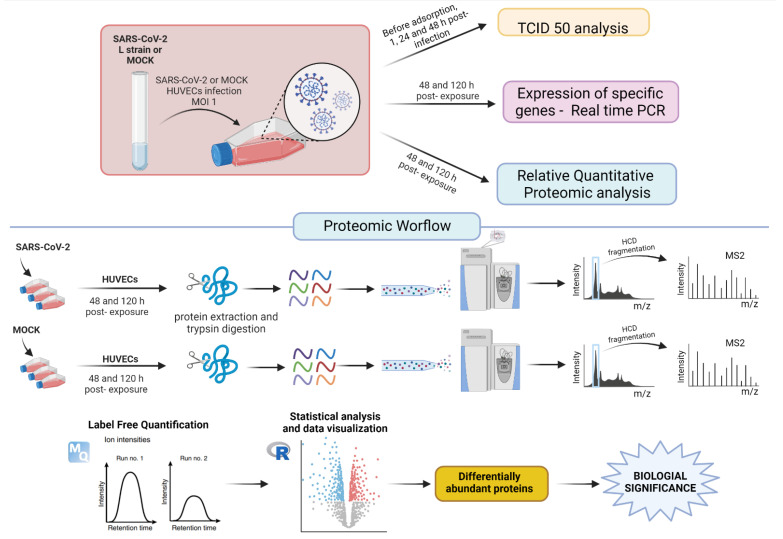
Shematic showing an experimental workflow for studying the response of HUVECs to SARS-CoV-2 L strain.

The experimental workflow performed in the present study is depicted in Figure 1. All analyses were based on the comparison between HUVECs exposed to SARS-CoV-2 and mock-infected cells.

### 2.1. SARS-CoV-2 Does Not Infect HUVECs

**Figure 2:**
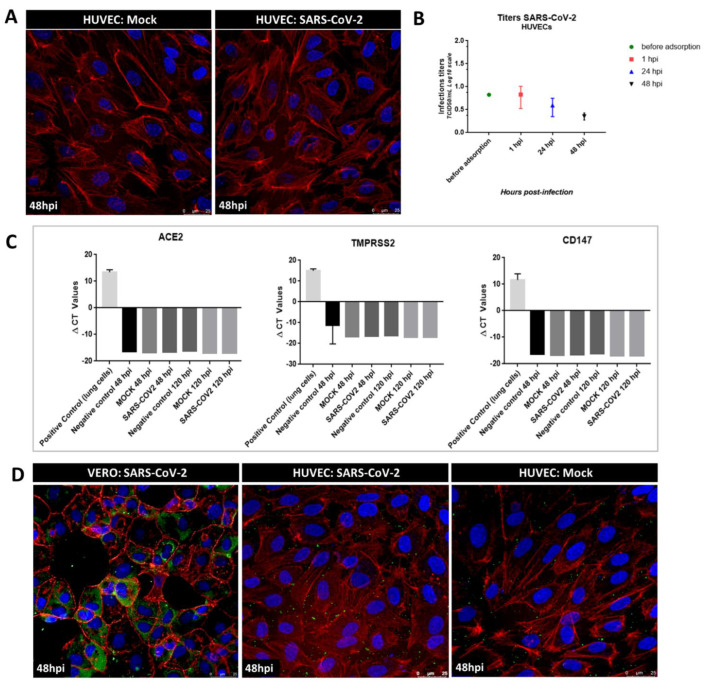
**Absence of SARS-CoV-2 productive infection in primary HUVECs.** HUVECs were infected with SARS-CoV-2 at MOI of 1 or mock-infected for 1 h and were cultured for up to 48 h. (**A**) Representative confocal microscopy images of HUVECs at 48 h after exposure show the integrity of the actin cytoskeleton without alterations. (**B**) Viral titer measurement by TCID_50_ assay of HUVECs culture after the indicated infection time showed a decrease in titer over time. Values shown are the means ± standard deviation (SD) of three independent biological samples. (**C**) RT- qPCR analysis shows no mRNA expression of *ACE2*, *TMPRSS2*, or *CD147* in primary HUVECs. Lung cells (HK2) and water were used as positive and negative controls, respectively. Bar charts show the delta C_T_ values of the three RT- qPCR measurements. (**D**) Representative confocal microscopy images showing the negative immunostaining profile of the viral nucleoprotein in HUVECs infected with SARS- CoV-2 at MOI of 1 or with mock and the positive immunostaining profile in Vero-E6 cells infected with SARS-CoV-2 at MOI of 0.001 at 48 hpi. In confocal images, **red** depicts actin stained with Alexa Fluor 647 phalloindin, **blue** depicts nuclei stained with Hoechst 33342, **green** depicts nucleoprotein stained with recombinant anti-SARS-CoV-2 nucleocapsid protein antibody indirectly labeled with secondary antibody anti-human IgG antibody conjugated with FITC.

To evaluate whether the primary endothelial cells were infected with SARS-CoV-2, we first analyzed cell morphology and measured the viral titers of HUVECs exposed to SARS-CoV-2 at MOI of 1. There were no visible cytopathic effects even 48 h post-virus exposure (Figure 2A), and the SARS-CoV-2 titer decreased at different time points (Figure 2B). As SARS-CoV-2 infection is highly associated with the expression of cell receptors, such as ACE2 and TMPRSS2, we measured the RNA expression of these receptors and *CD147*, a putative internalizing receptor of SARS-CoV-2. The RNA levels of the three receptors were negative in both mock- and SARS-CoV-2-exposed cells (Figure 2C). In addition, at the protein level, ACE2, TMPRSS2, CD147, or viral proteins could not be identified in the shotgun proteomic analysis of SARS-CoV-2-exposed samples (Supplementary Table S2). Confocal microscopy analysis showed negative staining for viral nucleoprotein (N), indicating that no viral proteins were produced in HUVECs, in contrast to the positive staining observed in the Vero cells used as a positive control (Figure 2D). Overall, these results indicate that the cells were not infected, and that the virus did not undergo replication cycles.

### 2.2. Proteomic Analysis

**Figure 3:**
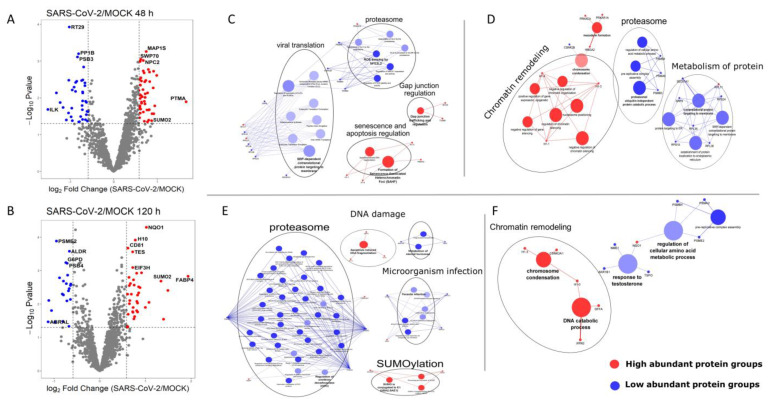
Functional clustering and enrichment analysis of differentially abundant proteins in HUVECs in response to SARS-CoV-2 exposure. (**A**,**B**) Volcano plots of the relative quantified protein groups showing a significantly high abundance of proteins in red (log_2_Fc ≥ 0.58 and −log_10_ *p*-value ≥ 1.12 = *p*-value ≤ 0.05) and low abundance proteins in blue (log_2_Fc ≤ −0.58 and −log_10_ *p*-value ≥ 1.12 = *p*-value ≤ 0.05). The gene names depicted in each plot were from proteins that presented the highest fold change or the lowest *p* values (*p*-value ≤ 0.001). Network representation of the enriched reactome pathways of the high (red) or low abundant (blue) protein clusters of SARS-CoV-2-exposed HUVEC samples after 48 h (**C**) or 120 h (**E**). Network representation of the enriched GO terms for biological processes of high (red) or low abundant (blue) protein clusters of SARS-CoV-2 exposed samples at 48 h (**D**) or 120 h (**F**). Nodes depict the enriched terms for pathways or biological processes. The connections between the nodes represent the shared proteins among them. Dots represent the main proteins associated with each biological process or pathway. The size of the nodes was directly proportional to the significance of the terms (corrected *p*-value ≤ 0.05, right-sided hypergeometric test, Bonferroni step-down FDR correction). Clustering and labeling of related GO biological processes or Reactome pathways were performed using AutoAnnotate. Black circles highlight enriched terms that indicate oxidative stress and/or endothelial dysfunction.

To investigate changes in the proteome of HUVECs, cells were exposed to SARS-CoV-2 or mock for 1 h. Across all replicates, 1490 and 1426 protein groups were quantified in HUVEC samples after 48 (Figure 3A and Supplementary Table S3) and 120 h (Figure 3B and Supplementary Table S4), respectively. Proteins with a *p* value ≤ 0.05, and an FC cut-off of 1.5 were considered to be differentially abundant. After 48 h, eighty-nine proteins met this criterion (Figure 3A, blue and red dots) and, after 120 h, sixty proteins (Figure 3B, blue and red dots).

Prothymosin alpha (PTMA) and integrin-linked kinase (ILK) were the most affected proteins at 48 h (Figure 3A). PTMA showed a 3.2-fold increase in abundance in SARS-CoV-2-exposed cells (log_2_FC = 1.664; Figure 3A; Supplemental Table S3). This protein is involved in a variety of cellular processes, including resistance to opportunistic infections and antiviral activity [24]. ILK showed a 2.8-fold decrease in abundance (log_2_FC = −1.5) in the SARS-CoV-2 exposed cells. ILK is a multifunctional protein that combines the functions of scaffold and signal transduction proteins [25]. Abrasion of the blood vessels triggers a decrease in ILK expression, leading to smooth-muscle cell migration and proliferation, which form a thickened neointima [25].

Of the DA proteins, only six had a *p*-value ≤ 0.001 after 48 h (Figure 3A, Table S1). Among them, three proteins, microtubule-associated protein 1S (MAP1S), switch-associated protein 70 (SWP70), and NPC intracellular cholesterol transporter 2 (NLTP/NCP2) were positively modulated. The other three proteins, mitochondrial 28S ribosomal protein S29 (RT29, also known as DAP-3: death-associated protein 3), serine/threonine-protein phosphatase PP1-beta catalytic subunit (PP1B), and proteasome subunit beta type-3 (PSB3) were found to have decreased abundance.

These proteins act on distinct cellular processes, including cytoskeletal organization, cellular metabolism, protein degradation, and cellular response to stress. It is important to note that SWP70 is a guanine exchange factor (GEF) that is localized in the cell membrane, regulates actin filaments, and is highly expressed in endothelial cells [26]. It has been observed to play a role in the endothelial activation of vGPCR-driven endothelial permeability in Kaposi’s sarcoma (KS), a disease caused by human herpesvirus 8 (HHV8) [27].

Fatty-acid-binding protein 4 (FABP4) and Costars family protein ABRAL (also known as ABRA C-terminal-like protein, ABRACL) were the most affected proteins after 120 h (Figure 3B). FABP4 showed a 3.8-fold increase in expression (log_2_FC = 1.93; Supplementary Table S4) in the SARS-CoV-2-exposed cells. It is an adipogenic protein that promotes angiogenesis in vein endothelial cells and plays a role in various pathological conditions such as atherosclerosis, cancer, insulin resistance, obesity, and hypertension [28,29]. FABP4 is also thought to be involved in neointimal hyperplasia after vascular injury through endothelial dysfunction and proinflammatory cytokine production [30].

ABRACL exhibited a 5-fold decreased expression in SARS-CoV-2-exposed cells (log_2_FC = −2.4, Supplementary Table S4). It is a low-molecular-weight protein with high sequence homology to the C-terminal domain of the actin-binding Rho activation protein (ABRA).

ABRACL proteins act as regulators of actin cytoskeleton dynamics and cell motility [31]. In cancer cells, downregulation of ABRACL suppresses proliferation, invasion, and migration [28]. ABRACL expression disrupts the balance between cellular F- and G-actin, and depletion of ABRACL expression can lead to increased actin nucleation and negative regulation of actin filaments [31].

Of the DA proteins, nine had a *p*-value < 0.001 after 120 h (Figure 3B, Table S3). Five of them, NAD(P)H dehydrogenase [quinone] 1 (NQO1), H1.0 histone, CD81, TES tetraspanin family member (TES) and eukaryotic translation initiation factor 3 subunit H (EIF3H), were positively modulated. Four proteins, proteasome activator subunit 2 (PSME2), proteasome subunit beta type-4 (PSB4), glucose-6-phosphate dehydrogenase (G6PD) and aldo-keto reductase family 1 member B1 (ALDR), showed decreased expression in SARS-CoV-2-exposed HUVECs. Taken together, these proteins are associated with endothelial dysfunction, indicating impairment of the ubiquitin–proteasome system (UPS). Moreover, endothelial CD81 is a marker of early human atherosclerotic plaques and facilitates monocyte adhesion before the onset of inflammatory response [32,33]. CD81 combines the ability to increase monocyte adhesion to non-inflamed endothelium with a specific upregulation in the endothelium of early atherosclerotic lesions [32]. It is also important to highlight the decrease in G6PD levels in HUVECs following viral exposure. Deficiency of this protein has been associated with a higher susceptibility to coronavirus infection and the severity of COVID-19 [34]. This protein is important for the maintenance of redox homeostasis, and a lack of G6PD has been implicated in several hematological disorders [35,36].

To better understand the relationship between the detected DA proteins and cellular mechanisms, we performed enrichment analysis of cellular pathways and biological processes. At both the time points, proteasome proteins were negatively regulated (Figure 3C–E). Among the DA proteins at 48 h post exposure, viral transduction proteins showed enrichment in low-abundance proteins (Figure 3C). Chromatin remodeling, senescence, and apoptosis-regulating proteins were predominantly positively regulated after 48 h (Figure 3D,F). The SUMOylation pathway was found to be enriched after 120 h (Figure 3E). Thus, enrichment analyses of Reactome-based pathways and GO-based biological processes revealed a significant decrease in the protein degradation machinery of cells (proteasome, metabolism of proteins) and antiviral responses (viral translation, microorganism infection), possibly due to an increase in SUMOylation of proteins.

We also examined the enrichment pathways and biological processes of proteins identified exclusively in samples exposed to SARS-CoV-2 or mock (Supplementary Figure S1). These results clearly showed a distinct profile of enrichment processes in SARS-CoV-2 samples (Figure S2A,B,E,F versus Figure S2C,D,G,H) and highlighted the differences between 48 and 120 h of exposure (Figure S2C,D versus Figure S2G,H)

**Figure 4:**
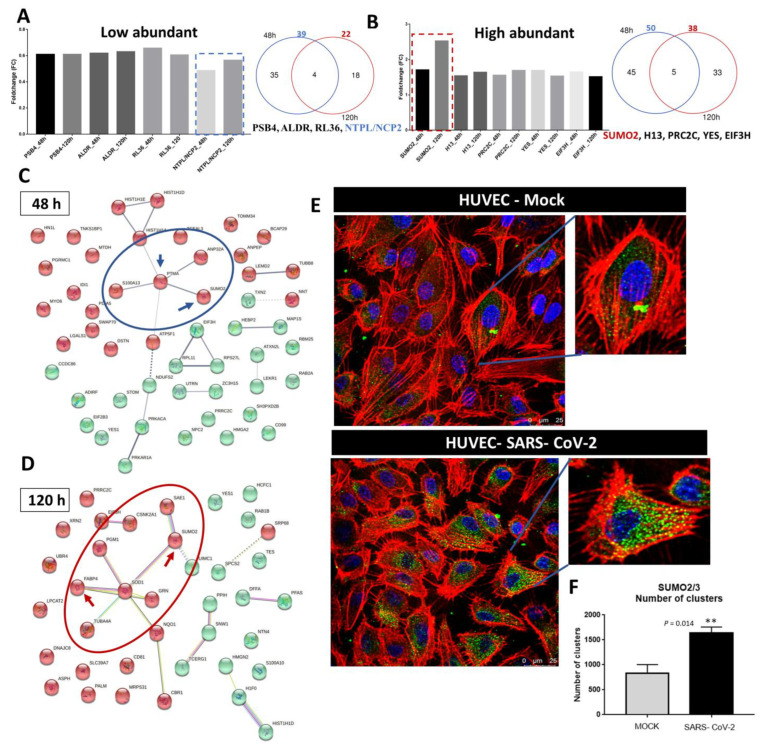
Increase in SUMO2 abundance upon SARS-CoV-2 exposure in HUVECs. (**A**,**B**) Bar chart and Venn diagram showing common differentially abundant proteins at both time points. The dotted blue box highlights the decrease in NTPL/NCP2 abundance over time and the dotted red box highlights the increase in SUMO2 abundance over time. Network of protein–protein interaction analysis using the K-means cluster of the highly abundant proteins at 48 h (**C**) and 120 h (**D**). Blue circles and arrows highlight the link between SUMO2 and PTMA. The red circles and arrows highlight that SUMO2 and FABP4 are clustered together. Green and red dots represent the cluster divisions based on K-means performed using STRING functional protein association networks. (**E**) Representative confocal microscopy images showing increased immunostaining for SUMO2/3 in virus-exposed HUVECs compared with mock-exposed cells after 48 h. Zoomed-in panels highlight the stronger staining of SUMO2/3 in SARS-CoV-2 exposed cells and (**F**) bar chart showing SUMO2/3 clusters in virus- and mock-exposed cells (*p*-value < 0.05, U-test Mann–Whitney. ** *p* ≤ 0.01) **Red** depicts actin stained with Alexa Fluor 647 phalloindin, **blue** depicts nuclei stained with Hoechst 33342, **green** SUMO2/3 stained with anti-SUMO2 + SUMO3 antibody, indirectly labeled with secondary antibody anti-mouse IgG conjugated with Alexa Fluor Plus 488.

We then examined which DA proteins were common at both of the studied time points. Nine DA proteins were present in both the 48 and 120 h samples (Figure 3A,B and Figure 4A,B). Four of them, ALDR, PSB4, NTPL, and RL37, showed a decrease in abundance after virus exposure (Figure 4A) and a higher abundance of five proteins, Small ubiquitin-related modifier 2 (SUMO2), Proline-rich and Coiled-coil-containing protein 2C (PRC2C), Eukaryotic translation initiation factor 3 subunit 3 (EIF3H), Tyrosine-protein kinase Yes (YES), and Histone H1.3 (H1.3), was detected in response to virus exposure (Figure 4B). SUMO2 was the only protein among the highly abundant proteins that showed a prominent increase in abundance over time (Figure 4B).

Immunofluorescence staining and confocal microscopy revealed the positive cytoplasmic staining of SUMO2/3 proteins in SARS-CoV-2-exposed cells in comparison to mock-exposed cells at 48 h (Figure 4D–F), confirming the proteomic findings. Counting of the 3D objects and measurement of fluorescence intensity showed that the number of SUMO2/3 clusters was more than doubled in the SARS-CoV-2 exposed samples, with no difference in cluster volume or average intensity (Supplementary Figure S2). Protein–protein network analysis of DA proteins showed an association between SUMO2 and PTMA and revealed that SUMO2 and FABP4 clustered together, which were the proteins that had the highest fold change among DA proteins at 48 and 120 h, respectively (Figure 4C,D). Moreover, SUMO-activating enzyme subunit 1 (SAE-1) was found to be differentially abundant at 120 h (log_2_FC = 0.61, Figure 4D) and was shown to be associated with SUMO2 in the network analysis. SAE-1 acts as an E1 ligase of SUMO1, SUMO2, and SUMO3, and is involved in the protein SUMOylation pathway.

## 3. Discussion

COVID-19 has been characterized as a thromboinflammatory disease. The more severe pathophysiology of COVID-19 often leads to impaired hemostatic balance and endothelial dysfunction [20,37]. To date, only a few studies have described the molecular mechanisms underlying endothelial dysfunction.

The entry of SARS-CoV-2 into host cells is associated with the interaction of the receptor-binding domain (S1-RBD) of the S protein with the ACE2 receptor on the host-cell membrane. In addition, crucial proteolytic processing of the S2-RBD cleavage site of the S protein is necessary for viral endocytosis [12,38]. Cell-surface proteases such as TMPRSS2 are responsible for this process [13]. It has been suggested that the differential susceptibility of endothelial cells from different tissues to SARS-CoV-2 infection correlates with the low expression or absence of the ACE2 receptor [39]. However, other cell surface molecules are under investigation as possible receptors for SARS-CoV-2 entry, such as CD147 [17,40].

Our results showed that ACE2, TMPRSS2, and CD147 were not expressed in HUVECs. These data corroborate the transcriptomic and epigenomic data indicating that the ACE2 receptor is not expressed in human endothelial cells [16,39]. In contrast, another study showed that HUVECs were positive for ACE2 and TMPRSS2 proteins (HUVECs, ATCC^®^ PCS-100-013 ™^;^ sex unknown) and described the infection of HUVECs at MOI of 2 and 5. However, viral replication was not observed in HUVECs, even at these MOIs [41]. This is in accordance with the absence of morphological changes, decrease in viral titers, lack of SARS-CoV-2-N protein staining, and lack of structural or accessory viral proteins described in our results, indicating that there was no productive infection in these cells. Our data are consistent with evidence suggesting that vascular endothelial cells are resistant to SARS-CoV-2 infection [16,39,42]. In fact, viral detection assays and immunohistochemical analysis of the SARS-CoV-2 N protein showed the inability of SARS-CoV-2 to infect primary endothelial cells from distinct tissues [43].

The first studies of possible endothelium infection were based on the presence of endothelial lesions, thrombosis, marked infiltration of inflammatory cells, and putative detection of viral particles in the endothelial cells of lung biopsies from post-mortem COVID-19 patients [44,45]. However, it is unclear whether this infection is specific to endothelial cells or occurs within the perivascular compartment [46]. There have been many advances in the recognition of the ultrastructure and morphogenesis of SARS-CoV-2 viral particles, suggesting misidentification of viral particles in these initial studies [23,46]. Moreover, endothelial dysfunction observed in lung biopsies was not proven to be a direct effect of SARS-CoV-2 infection. Of note is that our results were obtained from HUVECs exposed to strain L of SARS-CoV-2; the virus has accumulated mutations overtime and the possibility that other strains might induce distinct endothelial cell responses as well as different viral tropism cannot be ruled out.

Several proteomic analyses have been performed on distinct biological samples, mainly plasma and serum, from COVID-19 patients to identify disease signatures and targets for treatment [23]. Few proteomic studies have been conducted on cells, and this is the first report of a proteomic analysis of the HUVECs response upon exposure to SARS-CoV-2. Pathway enrichment analysis of proteomic studies of SARS-CoV-2-infected cells highlighted the following important pathways: mitochondrial processes, RNA processing, spliceosome, proteasome, ubiquitin-mediated proteolysis, and cell cycle [47,48]. Some of these pathways resemble the enrichment profile observed in HUVECs, in which mitochondrial respiration and translation responses to calcium, proteasome, senescence, chromatin remodeling, and DNA damage were enriched in response to SARS-CoV-2 exposure. Moreover, these pathways suggest that the virus induces oxidative stress in endothelial cells. It is known that SARS-CoV-2 infection induces an increase in reactive oxygen species (ROS) and interferes with host antioxidant defenses [49]. The expression of hypoxia-inducible factor 1 α (HIF1α), which can be induced by ROS and nitric oxide (NO), is increased in HUVECs in response to the SARS-CoV-2 recombinant S protein [50]. Furthermore, excessive release of mitochondrial ROS is suggested to be an important initial step in the endothelial dysfunction induced by SARS-CoV-2 [51].

In general, it has been described that an external stimulus can interfere with the cellular redox system leading to an increase in proteins related to SUMOylation [52]. This may explain the increase in SUMO2, SAE1, and SUMOylation pathway enrichment observed in HUVECs exposed to SARS-CoV-2. SUMO2 belongs to the SUMO family and is a ubiquitin-like protein that promotes the SUMOylation of target proteins [53]. This post-translational process regulates several cellular processes, including DNA repair, response to mitochondrial stress, innate immune response, and antiviral defense [52,53,54]. SUMOylation of proteins is described in the early stages of viral infections that leads to the polyubiquitination of cytosolic receptors such as retinoic acid-inducible receptor gene I (RIG-I) and melanoma differentiation-associated protein 5 (MDA5), preventing their degradation through the ubiquitin–proteasomal system [55,56]. Recently published data suggests the importance of SUMOylation in the context of SARS-CoV-2 [57]. The non-structural protein Nsp5 of SARS-CoV-2 stimulates the expression of inflammatory cytokines through SUMOylation of the mitochondrial antiviral signaling protein (MAVS) [58].

The functional protein association network showed a link between SUMO2 and PTMA, the most modulated proteins, at 48 h. PTMA has pleiotropic cell activity and a dual role where, intracellularly, it acts as a mediator of survival and proliferation, and, extracellularly, it acts as a biological response modifier [24]. It is also described to mediate immune function by conferring protection of the oxidative stress and resistance to opportunistic infections, with potent antiviral activity [59]. Studies have shown that extracellular PTMA has effective HIV-1-inhibitory activity [60]. Proteomic analysis showed an increase in PTMA in CD8+ T-cells in plasma samples from patients with severe COVID-19 [61]. PTMA may protect T-cells during lymphopenia in COVID-19 and the administration of thymosin alpha- 1(Tα1), final product of PTMA, is suggested as a potential approach to protect effector T-cells during COVID-19 [61].

Our data demonstrated that the ubiquitin–proteasome system (UPS) is enriched among proteins that present a decrease in abundance in virus-exposed HUVECs, suggesting an endothelial cellular defense mechanism to disable SARS-CoV-2 replication. The UPS is known to be a key process for the efficient infection of coronavirus and regulation of the cell signaling of immune cells [62,63,64]. Nonetheless, these defense processes can cause endothelial dysfunction, as inactivation of the proteasome system can generate other types of cellular stress, such as endoplasmic reticulum stress [63,65].

Furthermore, the observed increase in FABP4, which is implicated in insulin resistance and atherosclerosis [66,67,68]; SWP70, which has been shown to play a role in endothelial activation [27]; and CD81, which facilitates monocyte adhesion and is a marker for early atherosclerotic plaques [32] together with the decrease in ILK, whose expression has been shown to be negatively correlated with neointima formation [25]; PP1B, which is positively correlated with cell adhesion and migration [69]; and ABRAL, which modulates actin dynamics in favor of increasing the relative content of F-actin [28,31], suggest endothelial dysfunction. It is worth highlighting that FABP4 was detected in high abundance in response to SARS-CoV-2. An increase in FABP4 levels contributes to inflammation and oxidative stress in distinct inflammatory diseases [70,71].

## 4. Methods and Materials

### 4.1. Production of the Active SARS-CoV-2 Bank

The isolated virus SARS-CoV-2/SP02/2020/BRA (GenBank accession number: MT126808.1) was kindly provided by the Laboratory of Clinical and Molecular Virology (LVCM) of the Institute of Biomedical Sciences (ICB) at the University of São Paulo (USP), which has created a network of virus distribution in Brazil supporting many scientific research studies in the country [72]. All viral manipulations were performed at the Biological Safety Level-3 (BSL-3) facilities located at ICB at USP following the World Health Organization (WHO) guidelines for handling SARS-CoV-2 specimens. Vero cells (CCL-81, ATCC, Manassas, VA, USA) were grown in 100 mL of VP serum-free medium (VP-SFM, Thermo, Waltham, MA, USA) in T225 culture flasks, and then infected with SARS-CoV-2 at a multiplicity of infection (MOI) of 0.05. After 72 h post-infection (hpi), the supernatant was collected and centrifuged to remove cellular debris. The clear supernatants after centrifugation were aliquoted and stored in sucrose, sodium phosphate, and glucose (SPG) preservation solution at −80 °C. Vero cells were used to determine the titer of the active virus bank following the median tissue culture infectious dose (TCID_50_/mL) assay [73,74]. For infection control (mock), conditioned media from Vero cells were produced as follows: cells were grown in a volume of 100 mL of VP Serum-Free Medium (VP-SFM, Thermo) in T225 culture flasks for 72 h. After 72 h of growth in the culture flasks, the supernatant was collected and centrifuged to remove cellular debris. Aliquots were stored at −80 °C and were used to treat HUVECs as mock controls.

### 4.2. Kinetic of SARS-CoV-2 Infection in HUVECs

Monolayers of endothelial cells (HUVECs) were inoculated with SARS-CoV-2 at an MOI of 1 and allowed to adsorb for one hour at 37 °C with 5% of CO_2_. After the incubation time, the inoculum was removed and basal medium was added. The inoculated cells were incubated for 48 h. The MOI of 1 for HUVECs was established in pilot experiments, where analyses of viability and time of infection were performed. The TCID_50_/mL of the HUVEC supernatants obtained at 1, 24, and 48 hpi was determined in Vero cells [74].

### 4.3. HUVECs Culture Conditions

Endothelial cells were obtained from human umbilical veins as previously described [75], with some modifications. Briefly, the HUVECs were isolated by digestion with collagenase type IV 0.2 mg/mL in phosphate-buffered saline (PBS) and were maintained in 25 cm^2^ tissue culture flasks pre-coated with 2% gelatin until they reached 80% confluence for establishing primary cell culture. Then, the viable endothelial cells were maintained in growth basal media, EBM-2 (CC-3156, Lonza, Walkersville, MD, USA) supplemented with the EGM-2 MV microvascular endothelial SingleQuots^TM^ kit (CC-4176, Lonza, Walkersville, MD, USA) containing 10% fetal bovine serum (FBS), hydrocortisone, human fibroblast growth factor (hFGF), vascular endothelial growth factor (VEGF), human long R63 insulin-like growth factor-1 (R3-IGF-1) ascorbic acid, human epidermal growth factor (hEGF), gentamicin, amphotericin-B, and heparin. The cells were obtained from different donors to create an endothelial cell bank. Subcultures of HUVECs were performed using a solution of 0.05% trypsin and 0.02% EDTA (25200-072, Gibco, New York, NY, USA). The primary culture was characterized by morphological analysis and immunostaining assays using an antibody binding to von Willebrand factor (vWF) at 1:500 dilution (sc14014, Santa Cruz Biotechnology, Dallas, TX, USA). In some experiments, 7 mg/mL polymyxin B (P4932, Sigma Aldrich, St. Louis, MO, USA) was used to rule out lipopolysaccharide interference. All cell cultures were maintained in an atmosphere of 95% humidity, 5% CO_2_ at 37 °C.

### 4.4. Inoculation of Endothelial Cells with SARS-CoV-2

HUVECs were plated in 6-well plates at a density of 2 × 10^5^ cells/mL for 72 h. Subsequently, the cells were exposed to SARS-CoV-2 at an MOI of 1 for 1 h for viral adsorption, the supernatant was removed, and endothelial cells were maintained in basal growth media (EBM-2, Lonza) for 48 and 120 h. As a negative infection control, HUVECs were subjected to the same treatment as the mock medium.

### 4.5. Gene Expression Analysis

HUVECs were exposed to SARS-CoV-2 as described above. Supernatants were removed after 48 and 120 h, cells were rinsed with phosphate-buffered saline (PBS), and 1 mL of Trizol (15596018, Invitrogen, Carlsbad, CA, USA) was added to each well and the resulting lysates were stored at −80 °C until RNA extraction. Total RNA was isolated and purified using the Illustra ^TM^ RNAspin Mini Kit (25-0500-72, GE Healthcare, Freiburg, DE, Germany) following the manufacturer recommendations. Total RNA was quantified using a NanoDrop ND-1000 spectrophotometer (5225, Thermo Fisher, Milwaukee, WI, USA). The cDNA was synthesized using 500 ng RNA template, oligo-dT, and random hexamer primers using the SuperScript ^TM^III First-Strand Synthesis SuperMix (18080-400, Invitrogen, Carlsbad, CA, USA) following the recommendations. For the real-time quantitative polymerase chain reaction RT-qPCR (Reverse Transcriptase Quantitative) Fast SYBR^TM^ Green Master Mix real-time PCR (4385612, Life Technologies, Carlsbad, CA, USA) and gene-specific primers were used. RT-qPCR was performed using a QuantStudio 3 Real-Time PCR System thermocycler (A28131, Thermo Fisher Scientific, Woodlands, SIN, Waltham, MA, USA). The conditions for the RT-qPCR reactions were: 40 cycles of 95 °C for 15 s and 60 °C for 1 min using gene-specific primers to measure the expression of the panel of genes (listed in Supplementary Table S1). In addition, mRNA expression levels were normalized to the mean Ct of ribosomal protein L37a (RPL37A), which was used as an endogenous control. Data were analyzed using the delta Ct method [76]. The genomic material from a human non-small-cell lung-cancer cell line, LC-HK2 [77], kindly provided by Dr. Glaucia Santeli (State University of São Paulo, Laboratory of the Department of Cell Biology and Development), was used as a positive control for gene expression analysis.

### 4.6. Sample Preparation and Liquid Chromatography Tandem Mass Spectrometry (LC–MS/MS) Analysis

The HUVEC supernatants were removed after 48 and 120 h and the cells were washed three times with PBS. Cells were harvested in 1 mL of 2 M urea solution containing 5 mM DTT and maintained at −80 °C until protein extraction. Cells were lysed on ice, subjected to ultrasonication (5 cycles: 30 s sonication/30 s on ice), and centrifuged at 15,000× *g* for 30 min. The sample supernatants were subjected to the FASP protocol [78,79] using 10 kDa filter units (Merck Millipore, Darmstadt, Hessen, DE, USA), and in-filter trypsin digestion was performed. Tryptic peptides were desalted using custom-made stage tips (SDB-XC membranes) and dried under vacuum using a centrifugal concentrator (22331, Eppendorf, Hamburgo, DE, USA). Samples were dissolved in 20 µL of aqueous buffer containing 0.1% formic acid and quantified, and 250 ng of each sample was subjected to an LC-MS/MS analysis on a nano-LC EASY 1200 (LC-030378, Thermo Scientific, Waltham, MA, USA) system coupled with a Q Exactive Plus (03893L, Thermo Scientific, Waltham, MA, USA) mass spectrometer at the Mass Spectrometry Unit of CENTD (Butantan Institute, Butantã, Brazil). Peptide mixtures of each sample were loaded onto an Acclaim–PepMap100 C18 trap column (3 μm particle size, 100 Å pore size, 75 μm internal diameter, and 20 mm column length) in line with an analytical Acclaim PepMap column (2 µm particle size, 100 Å pore size, 150 mm length, and 50 µm inner diameter) at a flow rate of 200 nL/min. The mobile phases, solvent A (0.1% *v/v* formic acid) and solvent B (80% *v/v* acetonitrile containing 0.1% *v/v* formic acid), were used in a linear gradient (5–30% B in 50 min, 30–60% B in 13 min, 60–100% B in 2 min, and a step of 100% B for 5 min). The mass spectrometer was operated in positive, data-dependent mode, in which one full MS scan was acquired in the *m*/*z* range of 300–1500 *m*/*z*, followed by MS/MS acquisition using higher energy collisional dissociation (HCD) of the seven most intense ions from the MS scan using a window width of 2.0 *m*/*z*. The dynamic exclusion duration was set to 60 s. For the survey MS scan, a target AGC value of 3 × 10^6^ and maximum injection time of 200 ms were set, whereas the target value for the fragmentation ion (MS/MS) was set to 2 × 10^5^ and maximum injection time of 120 ms.

### 4.7. Proteomics Data Processing

MS and MS/MS spectra were analyzed using MaxQuant version 1.6.14 [80] and searches were performed against a customized database including all coronavirus sequences, reviewed *Homo sapiens* sequences downloaded from UniProt (total of 20,845 sequences, downloaded on 10 January 2020), common mass spectrometry contaminants, and decoy sequences. The Andromeda search engine was set to detect specific tryptic peptides at a false discovery rate (FDR) of 0.01 and to perform label-free quantification (LFQ) with default parameters. Methionine oxidation and acetylation of protein N-termini were set as variable modifications, and carbamidomethylation of cysteine was set as a fixed modification. The identified and quantified protein groups were post-processed in R. Proteins identified as contaminants, reverse sequences, or identified only by site were filtered out. For protein significance analysis, data were used as input for the *limma* R package [81] and proteins that presented *p*-value lower than 0.05 and a fold change of 1.5 (−0.585 < log_2_ FC > 0.585) were considered to be differentially abundant (DA) comparing SARS-CoV-2 and mock samples. Pathway enrichment analysis was performed using the Reactome database (*p*-value ≤ 0.05) [82], and protein–protein interaction network analysis was performed using STRING [83]. Network enrichment analysis was performed using ClueGO [84] and AutoAnnotate [85] plugins in Cytoscape software 3.9.1 [86]. ClueGO presents enrichment analysis as networks, where biological processes or pathways are represented as nodes and *kappa* scores are represented as edges. For the enrichment analysis, we applied a hypergeometric test and Bonferroni step-down correction for multiple hypothesis testing, considering terms from both Gene Ontology (GO) and Reactome databases. All biological processes and pathways in the networks presented a corrected *p*-value < 0.05. CluePedia, a functionality of ClueGO, was used to visualize the main proteins associated with the significantly enriched biological processes or pathways. All other ClueGO parameters were maintained at default. To facilitate visualization, clusters of semantically related terms were created using AutoAnnotate.

### 4.8. Immunofluorescence Analysis Using Confocal Microscopy

HUVECs were plated at a density of 1 × 10^4^ cells/cm^2^ in 10-well plates (CELLview™ Slide) (543079, Griner Bio-One, Frickenhausen, BW, DE, Germany) precoated with 2% gelatin. The cells were cultured for 48 h, as previously described. Subsequently, the cells were exposed to SARS-CoV-2 at an MOI of 1 or mock medium for an adsorption period of 1 h. Cell supernatants were removed and endothelial cells were maintained in growth basal media EBM-2 (Lonza, Walkersville, MD, USA) for 48 h. After this period, cells were fixed with 4% paraformaldehyde for 1 h at 4 °C and then washed twice with PBS. Cells were permeabilized using 0.1% Triton X-100 (93443, Sigma-Aldrich, St. Louis, MO, USA) solution for 15 min at 4 °C, washed twice with PBS, and blocked with 5% bovine serum albumin (BSA) (9048-46-8, Sigma-Aldrich, St. Louis, MO, USA) at room temperature for 40 min. The cells were incubated overnight with anti-SUMO2/3 antibody (1:100 dilution) (Ab81371, Abcam, Waltham, MA, USA) or Anti-SARS-CoV-2 nucleocapsid protein antibody [1A6] (1:500 dilution) (Ab272852, Abcam, Waltham, MA, USA). The cells were then incubated for 1 h with a goat anti-mouse IgG antibody conjugated with Alexa Fluor Plus 488 (1:1000 dilution) (A32723, Molecular Probes, Carlsbad, CA, USA) or anti-human IgG antibody conjugated with FITC (1:1000 dilution) (F0132, Sigma Aldrich, St. Louis, MO, USA). For morphological analyses, an additional incubation of 30 min was performed with 3 U/mL of phalloidin conjugated with Alexa Fluor 647 to detect F-actin (A12380; Molecular Probes, Carlsbad, CA, USA). After washing with PBS, cell nuclei were stained with 0.1 mg/mL Hoeschst 33342 (H3570, Invitrogen, Carthage, MO, USA). Cells were scanned on the *x*-, *y*-, and *z*-axes using a confocal microscope (TCS SP8, Leica, Mannheim, BW, DE, Germany) with 63×/1.4. A 1.4 objective lens was used and laser excitation at 405, 488, and 638 nm were performed using the LAS X software 22.04 scientific volume image (SVI), Amsterdã, The Netherlands (Leica Microsystems). The images were deconvolved and performed a 3D volumetric analysis to obtain quantitative data as the number of clusters, volume, and intensities of SUMO2/3 proteins using Huygens Essential software 22.04 scientific volume image (SVI), Amsterdã, The Netherlands (Leica Microsystems). Indirect immunofluorescence was measured using the mean ± SD from at least three experiments. The obtained means were compared using U-test Mann–Whitney nonparametric on GraphPad Prism (GraphPad Software v8). *p*-values lower than 0.05 were considered significant.

### 4.9. Data Availability

All mass spectrometry proteomics data were deposited via the PRIDE partner repository [87] to the ProteomeXchange Consortium (http://proteomecentral.proteomexchange.org; accessed on 16 August 2022) under the identifier PXD036068.

## 5. Conclusions

In summary, our results suggest that HUVECs, in the presence of SARS-CoV-2, exhibit an initial pattern of oxidative stress that leads to endothelial dysfunction characterized by the dysregulation of specific proteins that are known to be involved in endothelial damage. Studies applying FABP4-specific inhibitors should be performed to evaluate the potential to alter HUVECs response to SARS-CoV-2 and prevent the observed deleterious effects. Moreover, we highlight the increase in SUMO2 and the enrichment of the SUMOylation of protein pathways as an important process to consider in the dysregulation of the endothelium in COVID-19. Further analysis is important to characterize the specific target proteins for SUMOylation and evaluate how this process might contribute to endothelial cell damage. A full understanding of HUVEC responses independent of viral infection leading to endothelial dysfunction will contribute to mitigating the severity of homeostatic disturbance in COVID-19.

## Data Availability

All mass spectrometry proteomics data have been deposited via the PRIDE partner repository [87] to the ProteomeXchange Consortium (http://proteomecentral.proteomexchange.org; accessed on 16 August 2022) under the identifier PXD036068.

## Supplementary Materials

The following supporting information can be downloaded at: https://www.mdpi.com/article/10.3390/ijms231810452/s1. https://zenodo.org/deposit/7063141; https://doi.org/10.5281/zenodo.7063141, Reference [88] is cited in the Supplementary Materials.
