# Supplementary material for: Proteomic Analysis Identifies Molecular Players and Biological Processes Specific to SARS-CoV-2 Exposure in Endothelial Cells
Source: Int J Mol Sci. 2022 Sep 9;23(18):10452. doi: 10.3390/ijms231810452 (PMC9500950; doi:10.3390/ijms231810452)
Supplement: Supplementary file 1 [file ijms-23-10452-s001.zip › ijms-1899840-supplementary (1).pdf]

International Journal of
Molecular Sciences

MDPI

Supplementary Figure S1

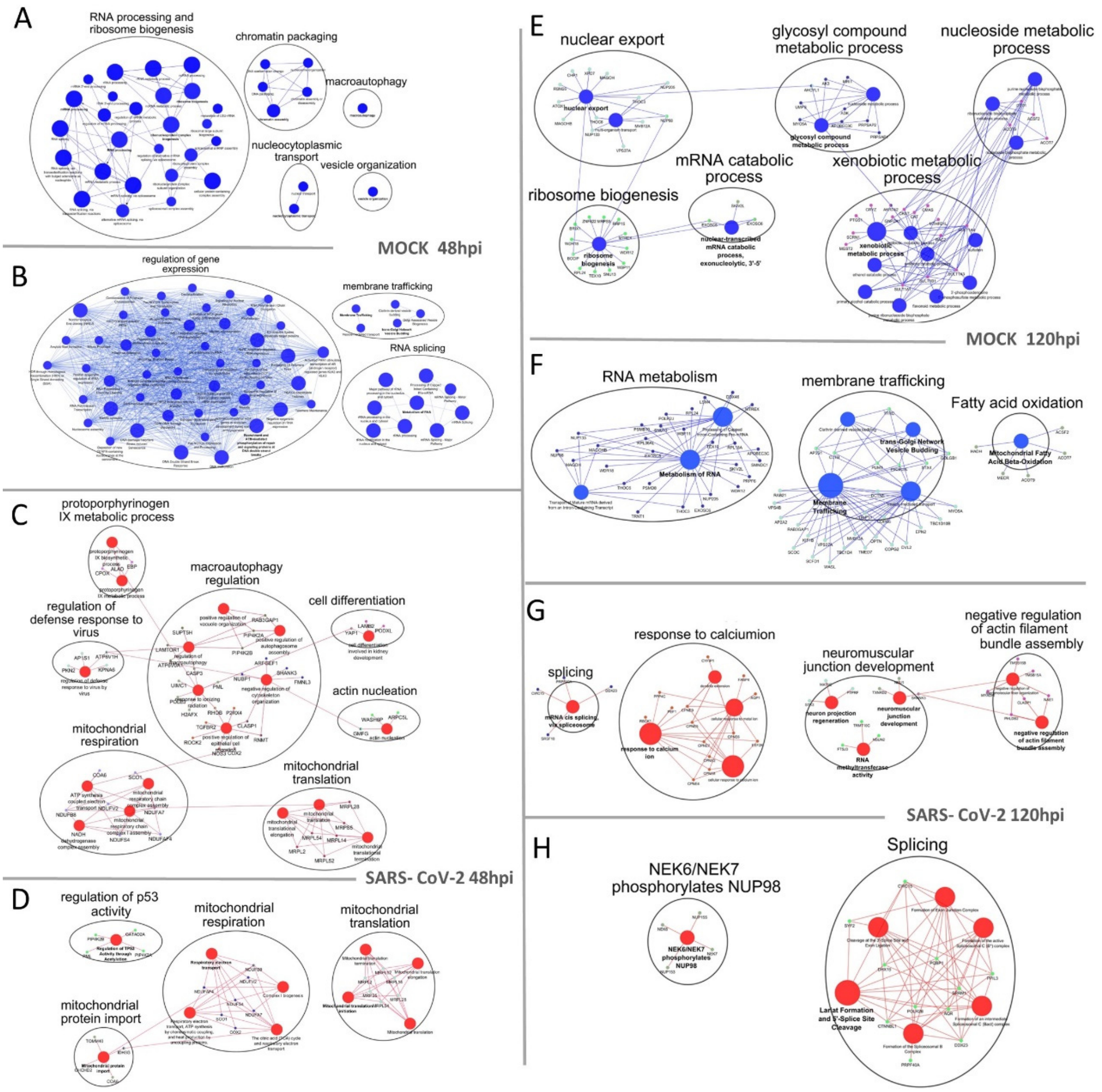

Suplementary Figure S1. Network representation of the enrichment of pathways and biological processes of proteins exclusively identified in HUVECs exposed to SARS-CoV-2 or mock infection. A global qualitative proteome analysis was performed to identify proteins in mock or SARS-CoV-2 exposed HUVECs using PEAKS software Studio X (Bioinformatics Solutions Inc.). The common proteins were excluded from this specific analysis, and proteins that were identified only in one of the studied conditions (control or virus-exposed) were subjected to enrichment analysis. Enriched Reactome pathways and GO biological processes of proteins were identified only in (blue) mock-exposed cells after (A and B) 48 h or (E and F) 120 h or in (red) SARS-CoV-2-exposed cells after (C and D) 48 h and (G and H) 120 h. Nodes depict the enriched terms for pathways or biological processes. The connections between the nodes represent the shared proteins among them. Dots represent the main proteins associated with each biological process or pathway. The size of the nodes was directly proportional to the significance of the terms (corrected p-value ≤ 0.05, right-sided hypergeometric test, Bonferroni step-down FDR correction). Clustering and labeling of related GO biological processes or Reactome pathways were performed using AutoAnnotate. Black circles indicate enriched terms that highlight enrichment terms.

Supplementary Figure S2

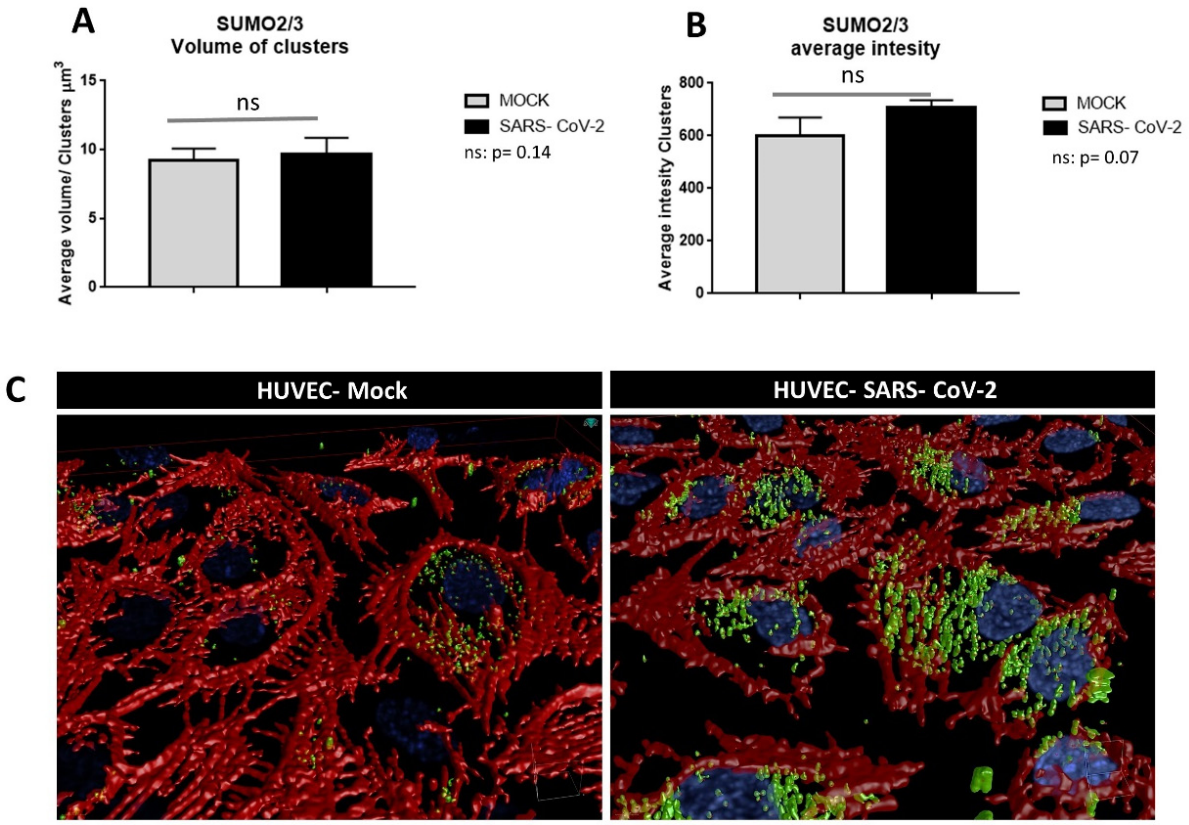

Supplementary Figure S2: Measurement of indirect immunofluorescence of SUMO2/3 protein in HUVECs exposed to SARS-CoV-2 for 48 h. (A) The volume of clusters and (B) the average intensity of clusters positively stained for SUMO2/3 were quantified through (C) 3D-confocal microscopy images of HUVECsexposed to mock or SARS-CoV-2. Green: SUMO2/3, indirectly labeled with secondary antibody anti-mouse IgG conjugated with Alexa Fluor Plus 488; red: actin stained with Alexa Fluor 647 phalloidin; blue: nuclei stained with Hoechst 33342. Cells were scanned in x, y and z axes using an SP8 confocal microscope with 63x/N.A 1.4 objective lens and laser excitation at 405, 488 and 638 nm using LAS X software 22.04 scientific volume image (SVI), Amsterdã, NL (Leica Microsystems). Indirect immunofluorescence was measured by mean ± SD of at least three experiments. Means were compared using the U-Test Mann–Whitney/nonparametric tests on the GraphPad Prism (GradPad Software v8). *p*-values ≤ 0.05 were considered significant.

Supplementary Table S1:

Primer sequences used for the expression analysis of SARS-CoV-2-internalizing cell receptors.

Target		Primer	Sequence	Refer
TMPRSS2		Forward	AATCGGTGTGTTCGCCTCTAC	[88]
		Reverse	CGTAGTTCTCGTTCCAGTCGT	
ACE2		Forward	AAACATACTGTGACCCCGCAT	[88]
		Reverse	CCAAGCCTCAGCATATTGAACA	
CD147		Forward	CCCAGGCAGTCGTGCTAGT	TC melo et al.
		Reverse	GGTGCCCTGTGACCTCTGT-	
***RPL37A* ***	NM_00	Forward	ATTGAAATCAGCCAGCACGC	NM_000998
		Reverse	AGGAACCACAGTGCCAGATCC	

* Reference of the endogenous genes that were used in the analyses.
